# CSCs: regenerating optimism for osteosarcoma treatment

**DOI:** 10.18632/oncotarget.25820

**Published:** 2018-08-03

**Authors:** Adam S. Levin

**Affiliations:** Department of Orthopaedic Surgery, The Johns Hopkins University, Baltimore, MD, USA

**Keywords:** osteosarcoma, cancer stem cells

During the last three decades, major advances have been made in the treatment of malignant neoplasms. Despite rapid progress in our understanding of bone sarcomas, however, oncologic outcomes have improved minimally since the landmark studies showing the critical role of chemotherapy in the treatment of osteosarcoma. It is possible that the small, incremental improvements that have occurred are attributable as much to advances in imaging as they are to greater effectiveness of systemic therapy. In fact, the most recent systemic treatment to substantially improve outcomes is muramyl tripeptide [1], which acts through a mechanism more akin to immunotherapy than to traditional cytotoxic chemotherapy. Unfortunately, this agent is unavailable in the United States.

Despite decades of ingenuity and investigation, the prognosis for a patient with metastatic osteosarcoma remains similar to that described by Meyers et al. in 1993 [2], and the prognosis still depends in large part on the ability to surgically eradicate all evidence of disease. In this context, the study by Crasto et al. [3] is a reason for renewed enthusiasm. Cancer stem cells (CSCs) represent a subpopulation of tumor cells that are thought to promote tumorigenesis. The pioneering work in CSCs began in acute myeloid leukemia, though more recent studies have suggested that small subpopulations of cells within solid tumors, including osteosarcomas, possess stem cell-like properties [4]. Furthermore, these subpopulations appear to be relatively radioresistant and chemoresistant [5] and therefore have been proposed as markers of poor prognosis. Neoadjuvant chemotherapy may help select for the CSC subpopulation due to their relative treatment resistance, which may explain the prognostic significance of tumor necrosis after neoadjuvant chemotherapy in osteosarcoma patients.

Elevated aldehyde dehydrogenase (ALDH) activity is associated with CSCs in both solid tumors and hematologic malignancies and has been similarly implicated in chemoresistance and tumorigenesis. Crasto et al. [3] used disulfiram as an ALDH inhibitor to target the CSC subpopulation in a murine osteosarcoma model. The authors showed a reduction in metastatic tumor burden after treatment with disulfiram, which was comparable to that achieved with doxorubicin chemotherapy. Interestingly, the enhanced proliferation and tumorigenicity of CSCs are more evident in vivo than in vitro, suggesting a dependence on the local niche [6]. Alternatively, this unexpected finding may reflect ALDH expression as being a marker, rather than a driver, of tumorigenicity. This dichotomy may help explain the lack of additive efficacy from treatment with disulfiram and doxorubicin together.

These favorable results using ALDH inhibition support renewed optimism that novel systemic therapies may provide additional therapeutic benefit for osteosarcoma via the targeting of CSCs to prevent metastatic disease. Moreover, the findings appear to have implications for other solid tumors beyond this rare disease. Potentially overlooked in the analysis by Crasto et al. [3], however, is an equally intriguing and exciting advance included in the analytical methods—the use of indocyanine green near-infrared fluorescence in the evaluation of osteosarcoma tumor burden. As noted by Crasto et al., the systemic treatments’ benefits of disulfiram and doxorubicin were largely in preventing the development of metastases, which is consistent with decades of human clinical results highlighting the role of complete surgical excision of the primary tumor in the treatment of osteosarcoma. Promising investigations regarding the utility of a fluorescent marker as a reliable imaging modality for the detection of viable tumor, as well as an in vivo modality to assess oncologic margins and residual tumor cells in the operative field, may prove these live imaging techniques to be as valuable in preventing local recurrence as CSC-targeted systemic therapy may be for preventing distant metastasis [7–9].
